# Embodied CO_2_ emissions of equity portfolios for Chinese asset managers

**DOI:** 10.1038/s41597-024-03308-x

**Published:** 2024-06-17

**Authors:** Jinglei Wang, Zengkai Zhang, Danbo Chen, Dabo Guan

**Affiliations:** 1 Institute of Blue and Green Development, Shandong University, Weihai, P. R. China; 2 State Key Laboratory of Marine Environmental Science and College of Environment and Ecology, Xiamen University, Xiamen, P. R. China; 3 School of Urban and Regional Science, Shanghai University of Finance and Economics, Shanghai, China; 4 Department of Earth System Science, Ministry of Education Key Laboratory for Earth System Modeling, Tsinghua University, Beijing, P. R. China; 5 The Bartlett School of Sustainable Construction, University College London, London, UK

**Keywords:** Environmental impact, Developing world, Sustainability

## Abstract

The 2015 Paris Agreement has set out the climate change target of limiting global warming to 1.5 °C, which poses a serious challenge to countries to reduce emissions. As the world’s largest carbon emitter, promoting the realization of the “dual-carbon” goal is the key to realizing China’s green transformation and high-quality development. Chinese asset managers play active roles in the capital market as an important channel of asset allocation. Currently, the vast majority of Chinese asset managers hold high percentages of high-carbon industries in their portfolios, and lack quantitative data of their carbon footprints embodied in equity investments, which faces huge carbon-related risks. Therefore, it’s an urgent need to comprehensively and scientifically measure financed emissions of Chinese asset managers, which is of great significance for asset managers’ carbon risk management and sustainable investment. This paper develops a detailed inventory of carbon emissions for equity portfolios managed by Chinese asset managers from 2010 to 2020, which stands as a pivotal reference for in-depth analysis of emission characteristics.

## Background & Summary

Climate change has resulted in extensive losses and profound repercussions, spreading from ecosystems to socioeconomic sectors^1^. The Paris Agreement sets forth an ambitious goal of curtailing the global average temperature rise to 1.5 °C above pre-industrial levels. Nonetheless, the trajectories proposed by the current Nationally Determined Contributions (NDCs) for emissions reduction seem poised to surpass this 1.5 °C goal^2,3^. Going green is being a global trend, China has committed to peak total carbon emissions before 2030 and achieve carbon neutrality before 2060. The “dual carbon” targets signify a profound shift towards a greener economy^4^. Asset managers occupy a pivotal position in this transformation, as they can champion Paris-aligned decarbonization by channeling capital to support transition activities^5^.

Climate change poses an extremely wide-ranging array of risks, which are acknowledged as a primary threat to asset managers^6^. While recent research has started to focus on increasingly potential carbon-related risks^7–9^, there remains severe lack of carbon emissions embodied in investment (financed emissions are categorized as Scope 3). This hampers asset managers’ ability to gauge the climate-related impact associated with investment activities^10^. Accurate and timely disclosure of the financed emissions is fundamental for comprehending carbon-related risks of portfolios and navigating emission reduction targets more effectively. Numerous institutions have provided methodologies to guide asset managers to measure their financed emissions, such as the Partnership for Carbon Accounting Financials (PCAF)^11^ and Task Force on Climate-related Financial Disclosures (TCFD)^12^. According to CDP 2022 report, Chinese asset managers lag far behind compared with their global counterparts, with none of them disclosing Scope 3 emissions data^13^.

To alleviate mechanisms for climate-related disclosures, the People’s Bank of China (PBoC), in collaboration with six other agencies, implemented Guidelines for Establishing the Green Financial System. This initiative has been instituted to standardize the climate disclosures of asset managers, without rules setting for financed emissions^14^. Additionally, the Ministry of Ecology and Environment (MEE) added climate-related information to the existing system for corporate environmental information, with a focus on CO_2_ emissions^15^. While China has made commendable strides in climate disclosure system, disclosure by asset managers is not yet mandatory and the availability of data is limited^16^. A notable issue is the voluntary and non-standardized nature of firm-level environmental disclosures at the firm level^17,18^. Contrary to the regulatory in developed countries, which require firms to disclose their CO_2_ emissions, in China, there is on a voluntary basis with only “optional disclosures” of carbon footprint details^19,20^. This absence poses challenges for asset managers, making it challenging to accurately gauge the genuine environmental impact of their investment entities due to information asymmetry^5^. To address the existing financed emissions data gap, this study introduces CO_2_ emissions inventories for 105 Chinese asset managers, focusing on their equity portfolios. We base our estimates on the carbon metrics recommended by TCFD. These metrics encompass three quantitative indicators: financed emissions, weighted average carbon intensity, and carbon emissions to revenue intensity. Moreover, this dataset encompasses 28 socioeconomic sectors, making it versatile for both emission patterns analysis and pinpointing carbon risk profile of asset managers.

## Methods

### Accounting scope

The World Resources Institute (WRI) and World Business Council for Sustainable Development (WBCSD) jointly developed the GHG Protocol Corporate Standard, which categorizes firms’ GHG emissions into three scopes^21^. Scope 1 covers direct emissions that companies have direct ownership or control over. Scope 2 is indirect emissions resulting from the consumption of purchased energy. Scope 3 refers to all other indirect emissions associated with both upstream and downstream activities in a company’s value chain. Scope 3 can be subdivided into 15 categories, notably including Purchased goods and services, Business travel, Transportation and distribution, as well as Investments, etc.

For asset managers, the most relevant category of Scope 3 emissions is financed emissions. This is category 15 on the Greenhouse Gas Protocol’s list of scope 3 emissions, and they’re the emissions linked to the investment activities of asset managers. Notably, financed emissions are, on average, 700 times greater than its direct emissions of asset managers. An asset manager’s Scope 3 emissions include the Scope 1, 2 and 3 emissions of its portfolio companies. However, quantifying these financed emissions can be intricate due to limited access to high-quality firm-level CO_2_ emissions data. In alignment with the PCAF, this study only considers the Scope 1 and Scope 2 emissions of investees, focusing on 105 asset managers in China.

### Data source

The list of Chinese asset managers is collected from Asset Management Association of China (AFCA), with a total of 143 fund firms and securities firms. We excluded the ones with incomplete equity portfolio data over the period of 2010 to 2020, and kept 105 asset managers left finally. The Chinese provincial multi-regional input-output tables are from CEADs database, which includes MRIO tables for 2012, 2015 and 2017 for Chinese 31 provinces. To fill gaps for missing years, we supplemented the data with figures from the adjacent year. Specifically, values of 2012 are used for 2010–2012; values of 2015 for 2013–2015; and values of 2017 for 2016 to 2020. For sectoral CO_2_ emissions, we collected multi-regional sectoral Scope 1 emissions from the Chinese provincial multi-regional carbon emissions from CEADs database, which includes 31 provinces of mainland China in 47 socioeconomic sectors. Scope 2 emissions are calculated based on the amount of electricity consumption and emissions factor. The electricity consumed is sourced from China Electric Power Yearbook. As for emission factor, we adopted an emission factor of 0.9402 ton CO_2_/mWh, derived from the weighted average of emission factors from regional power grids^22^. To address variances in sectoral classifications across databases, we merged into 28 sectors, as detailed in Table 1. We collected equity portfolios of 105 Chinese asset managers from S&P Capital IQ (Capital IQ) database. Capital IQ has detailed the equity portfolio of an asset manager including its shareholdings of the listed companies and updated quarterly since 2004, and we only chose the end of Quarter 4 of each year in this study. Additional details such as incorporation of state, SIC 4-digit code, and total revenue of investees are also obtained from Capital IQ, were prepared for the later region-sectoral allocations. In this study, we filtered out Chinese firms based on incorporation of state and then matched the SIC 4-digit code with the 28 aggregated sectors.

**Table 1:** Socioeconomic sectors.

Socioeconomic sectors	Category	Socioeconomic sectors	Category
Agriculture	The primary industry	Machinery	Heavy manufacturing
Coal Mining	Energy production	Transportation Equipment	Heavy manufacturing
Petroleum and Natural Gas Extraction	Energy production	Electric Equipment and Machinery	High-tech industry
Metals & Nonmetal Mining	Energy production	Electronic and Telecommunications Equipment	High-tech industry
Food Processing and Production	Light manufacturing	Measuring Manufacturing	High-tech industry
Textile Industry	Light manufacturing	Other Manufacturing	High-tech industry
Leather, Furs, Down and Related Products	Light manufacturing	Scrap and Waste	High-tech industry
Timber Processing, Bamboo, Cane, Palm Fiber & Straw Products	Light manufacturing	Production and Supply of Electric Power, Steam and Hot Water	Energy production
Papermaking and Paper Products	Light manufacturing	Production and Supply of Gas	Energy production
Petroleum Processing and Coking	Energy production	Production and Supply of Tap Water	Energy production
Raw Chemical Materials and Chemical Products	Heavy manufacturing	Construction	Construction
Nonmetal Products	Heavy manufacturing	Wholesale, Retail Trade and Catering Services	Service sector
Metals Smelting	Heavy manufacturing	Transportation and Telecommunication	Service sector
Metal Products	Heavy manufacturing	Others	Service sector

### Accounting method

The financed emissions in this dataset are estimated in accordance with the PCAF standard, aligning with the GHG Protocol Corporate Accounting and Reporting Standard. As referred, we quantified embodied CO_2_ emissions of equity portfolios using the equity share approach^23,24^. To illustrate, if E fund held a 10% equity share of China Energy Engineering Corporation Limited (SEHK:3996) in 2020, E fund would be responsible for 10% of its emissions spanning both Scope 1 and Scope 2.

According to the PCAF guidelines, financed emissions are calculated based on the attribution factor (AF), see Eq. (1) below.

1 $$Attribution\,facto{r}_{ct}=\frac{Outstanding\,amoun{t}_{ct}}{Total\,equit{y}_{ct}}$$

Where *AF*_*ct*_ refers to the proportional share of investment of the investee *c* in year *t*; *Outstanding amount*_*ct*_ represents the market value of the investee *c* which asset managers hold the equity proportion in year *t*; *Total equity*_*ct*_ refers to the sum of the market capitalization of the representative company *c* at the end of fiscal year *t*.

As a basis for investees’ CO_2_ emissions, we make use of an extensive sample of Chinese listed firms of equity portfolio spanning 2010 to 2020. Due to data gaps in firm-level CO_2_ emissions, one critical aspect of carbon accounting is selecting the appropriate emission factor to use. There are three main types of emission factors: activity-based, production-based, and economy-based approach. Activity-based and production-based emission factors are relatively precise and accurate approach associated with a specific activity, which needs higher quality fundamental data. While economy-based approach provides a rough estimate, the advantage of it is that it requires less detailed data than other methods. Therefore, when combined with total revenue, the corporate CO_2_ emissions can be expressed as:

2 $${E}_{ct}=\frac{{C}_{it}}{{O}_{it}}\ast {R}_{ct}$$

Where *E*_*ct*_ indicates the Scope 1 and Scope 2 emissions of investee c in year *t*. *C*_*it*_ refers to CO_2_ emissions (the sum of Scope 1 and Scope 2 emissions) by sector *i* in year *t*. *R*_*ct*_ indicates the total revenue of investee *c* in year *t*. *O*_*it*_ represents total output by sector *i* in year *t*. Note that Eq. (2) estimated the total CO_2_ emissions of invested company *c* in the year *t*.

### Investment carbon metrics

As aforementioned, financed emissions can be calculated by multiplying the attribution factor and corporate CO_2_ emissions. TCFD developed recommendations for more effective climate-related disclosures to promote better decision making^25^. In this study, we applied the TCFD framework to account for financed emissions and related carbon intensity indicators of equity portfolios; the equations can be expressed as (3) to (5).

This data is calculated by estimating the total emissions involved in each portfolio company’s activity, then applying an attribution factor, based on the investor’s holdings. The sum of these comprises the financed emissions.

3 $${F}_{t}=\sum _{c}A{F}_{ct}\ast {E}_{ct}$$

Where *F*_*t*_ refers to financed emissions in year *t*, which can be decomposed into Scope 1 financed emissions and Scope 2 financed emissions based on companies’ carbon scopes. *AF*_*ct*_ is attribution factor defined by the ratio of the outstanding amount based on its market value and the total equity of investee *c*. Financed emissions inform investment choices, are intended to facilitate the comparison of emissions across asset managers, provide a way for them to set emission reduction targets^26^, make net-zero commitments and help direct large investment flows alignment with Paris Agreement^27^.

4 $$WAC{I}_{t}=\mathop{\sum }\limits_{c}^{n}\left(\frac{{I}_{ct}}{{M}_{t}}\right)\ast \left(\frac{{E}_{ct}}{{R}_{ct}}\right)$$

Where *WACI*_*t*_ refers to Weighted Average Carbon Intensity, which Scope 1 and Scope 2 emissions are allocated based on portfolio weights; *I*_*it*_ is market value of investment on investee c in year *t*; *M*_*t*_ refers to total market value of its equity portfolio in year *t*; *R*_*ct*_ represents total revenue of investee c in year t. WACI can be more easily applied across different asset classes, such as bonds and project finance, since it does not rely on equity share approach. The Eq. (4) allows for portfolio sectoral decomposition and attribution analysis. For example, Electricity, Gas and Water contributed 61.56% to E fund’s WACI and Metal Smelting divestment could be attributed to a decrease of its WACI.

5 $$CER{I}_{t}=\frac{{\sum }_{c}^{n}{I}_{ct}/{M}_{t}\ast {E}_{ct}}{{\sum }_{c}^{n}{I}_{ct}/{M}_{t}\ast {R}_{ct}}$$

Where *CERI*_*t*_ refers to Carbon Emissions to Revenue Intensity, which normalizes investee c’s Scope 1 and Scope 2 emissions by the revenues apportioned to an investment based on equity share approach. In Eq. (5), the company’s revenue is used to adjust company size as a measurement of the efficiency of output. The metric takes into account differences in the size of companies and can be used to compare portfolios with another.

## Data Records

A total of 420 data records, including financed emissions, sectoral financed emissions, WACI and CERI are contained in the dataset. The present dataset is made public under Figshare (10.6084/m9.figshare.c.6936156.v3)^28^. Of these,

1. 105 are financed emissions inventory for asset managers (2010–2020) [File “Chinese asset managers’ financed emissions inventories, 2010–2020”];
2. 105 are WACI inventory for asset managers (2010–2020) [File “Chinese asset managers’ WACI inventories, 2010–2020”];
3. 105 are CERI inventory for asset managers (2010–2020) [File “Chinese asset managers’ CERI inventories, 2010–2020”];
4. 105 are sectoral financed emissions inventory for asset managers (2010–2020) [File “Chinese asset managers’ sectoral financed emissions inventories, 2010–2020”];

## Technical Validation

### Uncertainty

Uncertainty analyses are an important tool for improving emission inventories, which are crucial for addressing carbon-related risks and ensuring adequate funding for the transition towards carbon neutrality. The inventory uncertainties arise for various reasons. For example, corporate CO_2_ emission is calculated using average-sector carbon intensities (emission factors) multiplied by the total revenue. In this study, we adopted Scope 1 from CEADs multi-regional sectoral inventory dataset and Scope 2 is calculated based on the weighted average of the emissions factors archived by the NDRC. There are differences in the sectoral classifications of each database, therefore, we aggregated them into 27 sectors of Exiobase dataset, as shown in Table 2. It’s worth noting, however, that different datasets may have large variations in China’s CO_2_ emissions, primarily due to the uncertain emissions factors of China’s fossil fuel combustion. To provide a quantitative overview of sectoral carbon emissions, we distilled information from two sources: (1) For Scope 1 emissions, we drew from Chinese provincial multi-regional carbon emissions from CEADs (CEADs Provincial) and Exiobase dataset; (2) For Scope 2 emissions, we adopted an emission factor of 0.9402 tons CO_2_/mWh, derived from the weighted average of emissions factors from regional power grids in comparison with GTAP-E dataset.

**Table 2:** Socioeconomic sectors of Exiobase datasets.

Socioeconomic sectors	Category	Socioeconomic sectors	Category
Agriculture	The primary industry	Nonmetal Products	Heavy manufacturing
Coal Mining	Energy production	Metals Smelting & Products	Heavy manufacturing
Petroleum and Natural Gas Extraction	Energy production	Machinery	Heavy manufacturing
Metals Mining	Energy production	Electric Equipment and Machinery	High-tech industry
Nonmetal Mining	Energy production	Electronic and Telecommunications Equipment	High-tech industry
Food Processing and Production	Light manufacturing	Other Manufacturing	High-tech industry
Beverage and Tobacco	Light manufacturing	Transportation Equipment	Heavy manufacturing
Textile Industry	Light manufacturing	Scrap and Waste	High-tech industry
Garments and Other Fiber Products	Light manufacturing	Electric Power, Gas and Water	Energy production
Leather, Furs, Down and Related Products	Light manufacturing	Construction	Construction
Wood and wood products	Light manufacturing	Wholesale, Retail Trade and Catering Services	Service sector
Papermaking and Paper Products	Light manufacturing	Transportation and Telecommunication	Service sector
Petroleum Processing and Coking	Energy production	Other Services	Service sector
Raw Chemical Materials and Chemical Products	Heavy manufacturing		

**Fig. 1:**
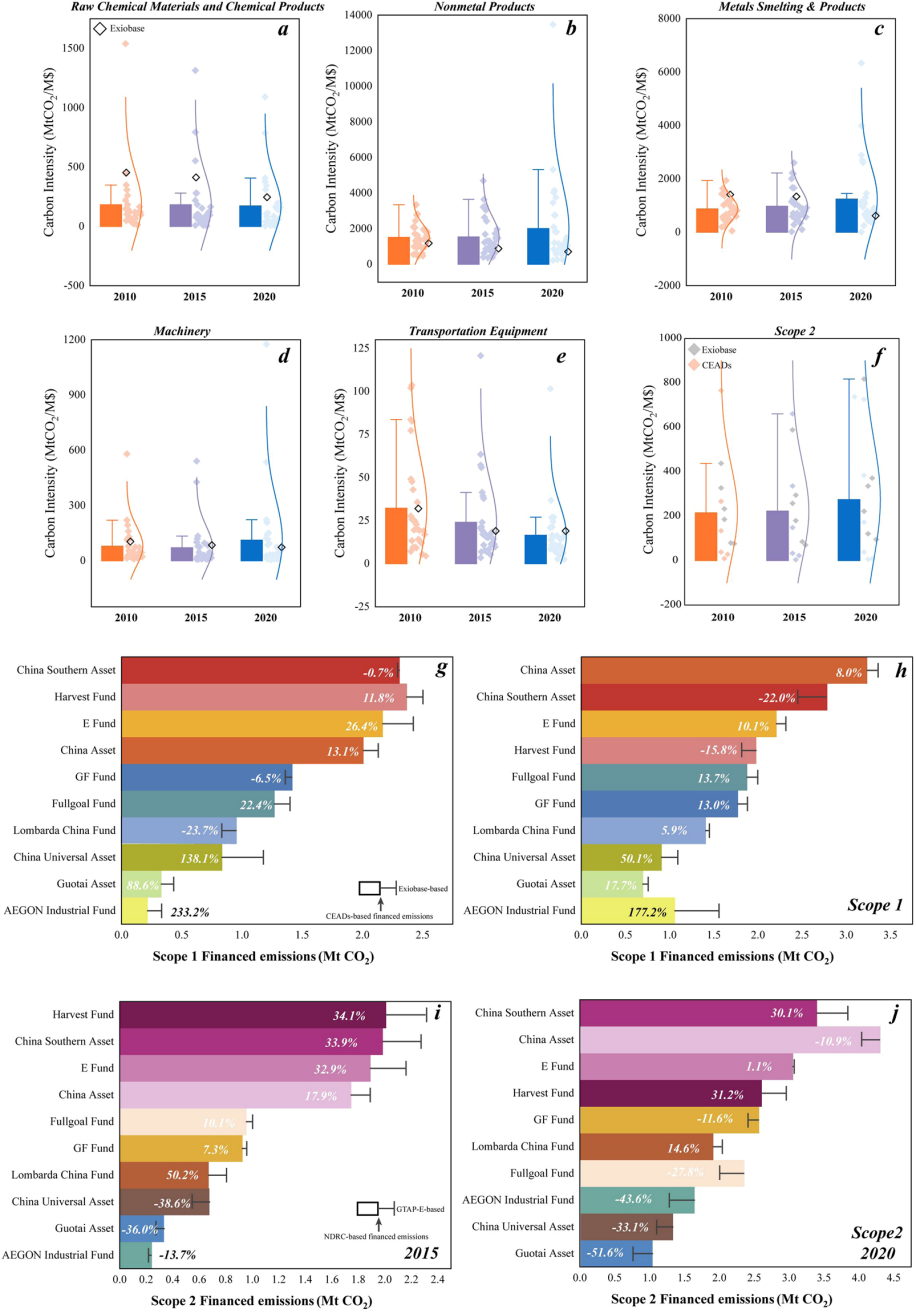
Comparison of carbon intensity and financed emissions among top 10 asset managers. (**a**–**f**) presents the comparison of CEADs’ Scope 1 emission intensity of key heavy sectors (Raw Chemical Materials and Chemical Products, Nonmetal Products, Metals Smelting & Products, Machinery, Transportation Equipment) and Scope 2 by provinces with Exiobase’s, respectively in 2010, 2015 and 2020. (**g**–**j**) represents Scope 1 financed emissions and Scope 2 financed emissions, respectively in 2015 and 2020.

There are some obvious strengths from our results in comparison to other prevailing data sources. From Fig. 1, we found that there were significant differences in carbon intensity among sectors, e.g., Scope 1 carbon intensity of Raw Chemical Materials and Chemical Products in 2020 was range from 3.27 tons CO_2_/M $ to 1091.39 tons CO_2_/M $ with a mean value of 170.09 tons CO_2_/M $, while Exiobase’s carbon intensity was 246 tons CO_2_/M $. Scope 1 carbon intensity of Nonmetal Products tended to be dispersed over the period 2010–2020, with the uncertainties from 216.12 tons CO_2_/M $ to 13471.86 tons CO_2_/M $ in 2020. The mean value decreased from 440.60 tons CO_2_/M $ in 2010 to 216.12 tons CO_2_/M $ in 2020; in contrast, the Exiobase’s value was significantly higher than CEADs Provincial mean value, which was 3.29 times higher in 2020. Comparing Scope 2 carbon intensity of the five high-carbon sectors based on NDRC and GTAP-E, carbon intensities of the NDRC approach were relatively high, e.g., the carbon intensity of the Metal Products in 2020 was 4.81 times; and the Machinery industry was 19.64 times that of the GTAP-E. Therefore, we applied the above carbon intensities to account for Scope 1 and Scope 2 financed emissions of the top 10 Chinese asset managers, respectively. Except for China Universal, Guotai Asset and AEGON Industrial Fund, the difference in Scope 1 financed emissions were basically within 25%; Scope 2 financed emissions calculated based on GTAP-E were significantly higher than those calculated by NDRC, with the uncertainties from 1.1% to 51.6%. The allocation of electricity was based on sectors, ignoring the region-crossing differences, but NDRC was relatively reliable compared to GTAP-E.

### Limitations

There are several limitations of our emission dataset. First, data quality is a major obstacle for asset managers to calculate financed emissions. Several factors contribute to this difficulty. The fact that Chinese listed firms don’t mandatorily disclose their CO_2_ emissions is a major contraint factor. Furthermore, annual releases of MRIO tables are rare. This absence necessitates the use of alternative methodologies to bridge gaps, often by interpolating from adjacent years, which inevitably compromises the dataset’s accuracy and reliability. We rely on economy-based emissions factors to estimate corporate CO_2_ emission in reporting year. This estimation can lead to inaccuracies, as it assumes that all goods and services have the same carbon intensity, which is not always the case. In the future, we will explore the self-reported CO_2_ emission data to update our dataset to analysis the entity specific. Secondly, we assume that all firms operating in the same industry have identical production technology, ignoring inter-firm differences. Specifically, the electricity sector, including wind, solar and thermal energy, should be distinguished by different emission factors. In our forthcoming research, we will further improve the accuracy of the datasets and allocate specific emissions factors with different types of firms.

## Data Availability

The Python Code used to generate the emission inventories is publicly available on GitHub (https://github.com/lareina678/Embodied-CO2-emissions-of-equity-portfolios-for-Chinese-asset-managers.git)^29^, and as an archive on Figshare.
